# Microbiota-based biomarkers of infection risk in patients undergoing vascular endograft implantation: a pilot study

**DOI:** 10.3389/fmed.2025.1680148

**Published:** 2025-12-10

**Authors:** Arianna Delicati, Elda Chiara Colacchio, Beatrice Marcante, Giulia Stinziani, Franco Grego, Luciana Caenazzo, Pamela Tozzo

**Affiliations:** 1 Legal Medicine Unit, Department of Cardiac, Thoracic, Vascular Sciences and Public Health, University of Padova, Padova, Italy; 2 Department of Pharmaceutical and Pharmacological Sciences, University of Padova, Padova, Italy; 3 Unit of Biostatistics, Epidemiology and Public Health, Department of Cardiac, Thoracic, Vascular Sciences and Public Health, University of Padova, Padova, Italy; 4 Vascular Surgery Unit, Department of Cardiac, Thoracic, Vascular Sciences and Public Health, University of Padova, Padova, Italy

**Keywords:** healthcare-associated infection (HAI), public health, infection prevention, vascular endograft, personalized medicine

## Abstract

**Background:**

Healthcare-associated infections (HAIs) remain a critical issue for healthcare systems worldwide, contributing significantly to increased morbidity, mortality, and healthcare costs. Post-surgical and device-associated HAIs are particularly concerning due to their severe and potentially fatal clinical outcomes. Although it remains largely unexplored, the interaction between host, implanted device, and human microbiota is increasingly recognized as a potential factor in HAI onset.

**Methods:**

In this context, the present project aims to investigate—through 16S rRNA gene sequencing—the potential predictive and pathogenetic role of the skin and oral microbiota in patients undergoing vascular endografts (VEGs) implantation. Microbial profiles of patients who developed HAIs (HAI group) were compared with those who did not experience any post-surgical infectious complications (NoHAI group) to identify risk biomarkers, dysbiotic microbial patterns, and potential evolutionary trajectories that predispose to the HAIs onset.

**Results:**

Oral samples from HAI patients showed reduced microbial diversity (Shannon index, *p*-value = 0.597), whereas skin samples showed a significantly higher diversity (*p*-value = 0.023) compared with NoHAI patients. Furthermore, specific taxa emerged as potential indicators of HAIs susceptibility, including the phylum Firmicutes D (*p*-value < 0.001), the genera *Staphylococcus* (*p*-value = 0.011) and *Haemophilus* D (*p*-value = 0.036), and the species *Prevotella denticola* (*p*-value = 0.01) and *Streptococcus mutans* (*p*-value = 0.005).

**Conclusion:**

These results provide preliminary insights into the microbiological dynamics that may predispose patients to the onset of infections. Although further validation on larger and more diverse surgical populations is needed, these microbial signatures could represent promising targets for future pre-operative risk stratification and personalized preventive strategies in surgical patients.

## 1 Introduction

Infectious diseases, defined as illness caused by pathogens or their toxic products, have profoundly influenced human, shaping societies, economies, and cultures. From the Black Death in the fourteenth century to the recent COVID-19 pandemic, infections have caused millions of deaths and changed the course of history (1–3). Healthcare-associated infections (HAIs), defined as infectious acquired during the course of healthcare treatment and manifesting at least 48 h after admission or within 30 days of a healthcare treatment (4, 5), remain a major public health concern. They not only threaten the health and safety of patients, but also represent a significant economic burden for health systems, through both the direct costs associated with prolonged hospitalization and treatment, and the indirect costs related to medical-legal litigation (6, 7). Over recent decades, their incidence has increased due to population aging and the growing number of immunocompromised patients. In Europe, according to the Point Prevalence Surveys (PPS) of HAIs coordinated by the European Centre for Disease Prevention and Control (ECDC), the mean prevalence of HAIs rose from 6 to 7.1% within ten years, corresponding to approximately 37,000 deaths annually (4, 8–10).

Among the different types of HAIs, those occurring in patients undergoing vascular endografts (VEGs) implantation require particular attention. VEGs, used to treat vascular diseases such as aortic aneurysms, have revolutionized surgery by reducing morbidity and mortality compared with open procedures. However, despite their clinical benefits, these patients remain at risk of serious infectious complications (11).

Traditionally, VEG-related infections refer to those directly involving the prosthetic device itself. Although these events are relatively rare (0.2–5% of cases), they are associated with high mortality rates, up to 30% (11–16). Beyond these prosthetic infections, however, patients who undergo VEGs implantations are also at risk of developing other HAIs (such as urinary, surgical-site, or pulmonary infections) that are not directly linked to the graft but arise in the post-operative period (11, 17). The incidence of HAIs in these cases is not well defined, yet any such infection may represent a potential risk factor for graft infection or postoperative complications. This highlights the need for innovative strategies for the prevention and management that consider the patient’s broader susceptibility to infections (11).

In this context, the present study focused on HAIs occurring in patients who underwent VEGs implantation, rather than infections of the implanted device itself. This population was selected because it represents a clinically homogeneous and well-defined surgical cohort, offering an ideal starting model to investigate the influence of host-related factors (such as the microbiota) on HAIs onset. Although this choice limits the generalizability of the finding to a broader surgical population, it enhances internal validity by minimizing variability related to surgical techniques and perioperative management, useful aspects for a preliminary study.

The human microbiota plays a critical role in maintaining human health, contributing to colonization resistance against pathogenic infections. Alteration in the microbiota, known as dysbiosis, can compromise this protective function, predisposing individuals to infections, including HAIs (18–20). Although HAIs are typically attributed to well-known opportunistic pathogens such as the “ESKAPE bacteria” (*Enterococcus faecium, Staphylococcus aureus, Klebsiella pneumoniae, Acinetobacter baumannii, Pseudomonas aeruginosa,* and *Enterobacter* spp.) and *Clostridium difficile*, recent studies have highlighted that a broader microbial community may predispose individuals to infections (10, 21, 22).

For this reason, we focused on two key microbial niches—the skin and the oral cavity which represent fundamental components of the body’s first line of defence and potential reservoirs or transmission interfaces for HAIs-related pathogens (23–27). The oral microbiota could be directly linked to systemic infections and has been implicated in bacteremia and endocarditis due to the translocation of oral bacteria into the bloodstream, especially in patients with vascular implants (28). Conversely, the skin microbiota, while could be influenced by the external environment through bidirectional interactions, also includes stable commensal communities that play a critical role in maintain barrier integrity and preventing pathogen colonization (23–25). Investigating both anatomical sites therefore allow to evaluate microbial factors contributing to infection susceptibility, providing a more comprehensive understanding of the possible microbial factors influencing susceptibility to HAIs in surgical patients.

Current estimates suggest that up to half of all of HAIs are preventable through strict adherence to standardized infection prevention and control measures in healthcare settings (10). Therefore, effective prevention strategies must extend beyond standard hygiene practices. A deeper understanding of the interaction between host microbiota, medical devices, and environment exposure may reveal early biomarkers of infection risk. Such knowledge can inform the design of targeted preventive strategies and personalized infection control measures that effectively reduce the burden of HAIs and their associated costs (23, 29–31).

In this context, this study aims to explore the composition and potential predictive value of the oral and skin microbiota in patients undergoing VEG implantation, as part of a broader effort to integrate predictive microbiological biomarkers into personalized medicine approaches. Such integrations may significantly enhance the prevention of HAIs, improve patient outcomes, and inform broader public health strategies. By tailoring preventive and therapeutic interventions to individual microbiota profiles, healthcare systems can implement more effective, patient-centered approaches to infection control, ultimately reducing the incidence of HAIs and lowering related healthcare costs.

## 2 Materials and methods

### 2.1 Ethical considerations

The study protocol was performed in accordance with the Declaration of Helsinki and received approval from the Territorial Ethics Committee of the Central-East Veneto Area with Protocol No. 0018838 on March 13, 2024. All participants provided written informed consent before their inclusion in the study. All personal data collected in this study were processed in compliance with the General Data Protection Regulation (GDPR, EU Regulation 2016/679). Data were pseudonymized before analysis, and access was limited to authorized researchers only. Participants were informed about data management, usage, and their rights, including the possibility to withdraw their consent at any time.

### 2.2 Sample collection

Samples collection was performed on patients who had undergone VEGs implantation. Patients were selected based on well-defined criteria, including age between 18 and 90 years, ability to provide valid informed consent, and absence of overt immune system diseases or neoplastic diseases. Patients who had taken antibiotics in the four weeks preceding sampling or who had developed HAIs in the six months before the implantation of VEGs were excluded.

Patient enrolment was performed with collaboration between the “Unità Operativa Complessa (U.O.C.) di Chirurgia Vascolare dell’Azienda Ospedale-Università di Padova (AOUPD)” and the Laboratory of Forensic Genetics of the “U.O.C. di Medicina Legale e Tossicologia, AOUPD.” After providing written informed consent, recruited patients were included in the study sample and classified into two groups: the “case” group (patients who developed HAIs after surgery) and the “control” group (patients who did not develop HAIs).

Given the limited sample size and the preliminary nature of the study, a “non-one-to-one matching” approach was deliberately adopted to preserve adequate representativeness of both HAI and NoHAI groups while maintaining balance in age and gender. In particular, a total of 20 patients, ranging from 64 to 88 years, were selected for the study and sampled. These patients were subdivided in 10 for the “case” group and 10 for the “control” group, including 8 males and 2 females per group. This design ensured a sufficient degree of comparability given the confirmed absence of other significant differences between groups in demographic or clinical collected variables except for hospitalization length (an expected consequence of infection), as described below in the results section.

Sample collection was performed, for each patient, 12 months after surgery, using non-invasive procedures. Samples were collected by rubbing sterile swabs on both palm of the patient’s dominant hand and patient’s oral cavity. A second analogous sampling, from just cited two body sites, was repeated one month later. This timing was intentionally selected to capture a stable microbiota configuration, minimizing transient post-surgical, hospitalization, and antibiotic-related perturbations, as well as seasonal and daily variability. Evidence from previous studies indicates that microbial communities tend to gradually re-equilibrate toward their individual baseline after major perturbations (32–34). Accordingly, this approach aimed to approximate the characteristics of the patients’ microbiota in configurations potentially associated with the pre-operative period, rather than during peri- or post-operative ones.

**Figure 1:**
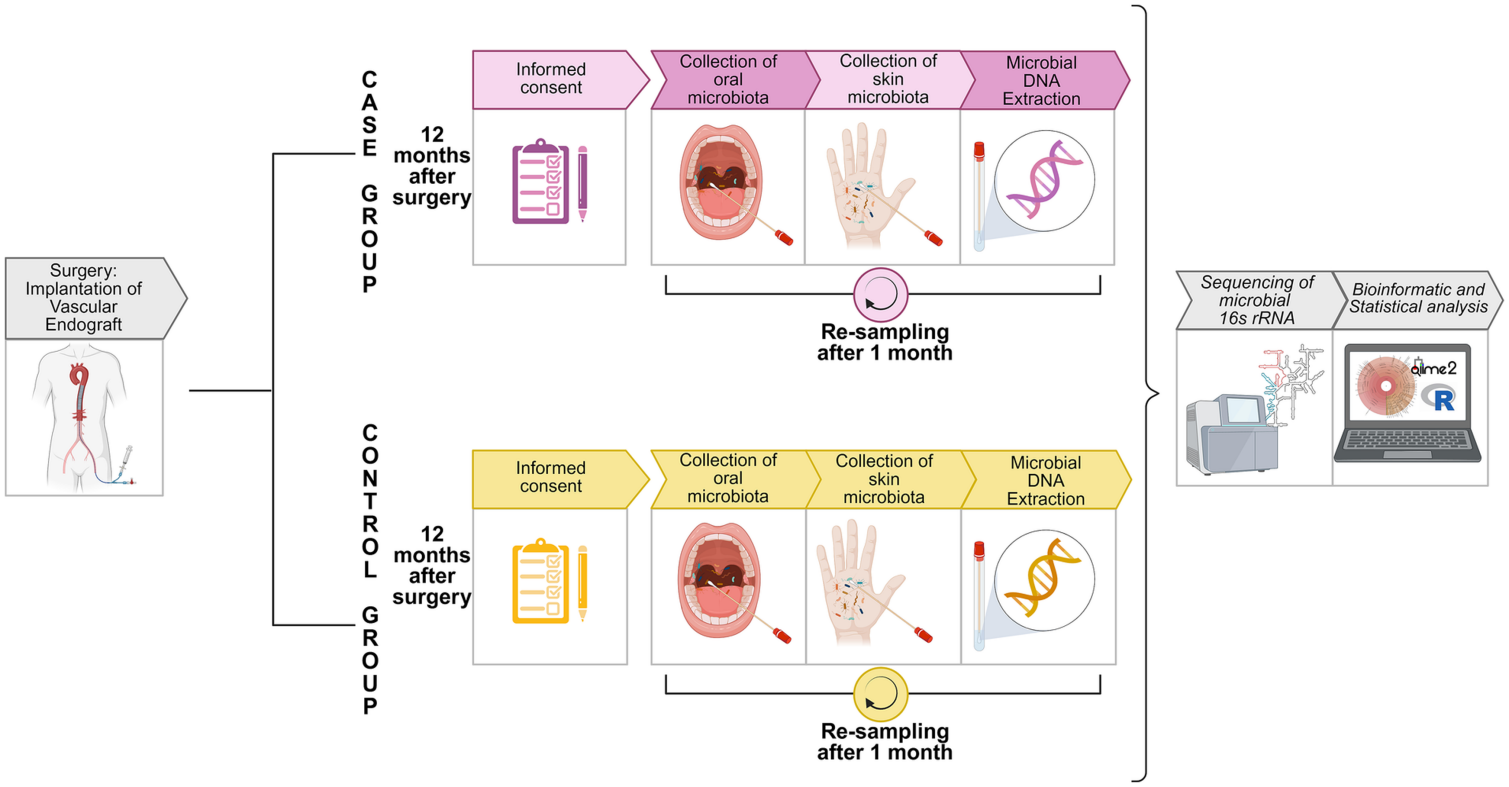
Experimental protocol overview. Schematic representation of the experimental workflow for the analysis of oral and skin microbiota in patients undergoing VEGs implantation. Patients were divided into two groups: the “case” group, if they developed an HAI after surgery, and the “control” group, if they did not develop an HAI after surgery. Created in BioRender https://BioRender.com/wvlf2lm (35). HAI, healthcare-associated infection; VEGs, vascular endografts.

To minimize inter-individual variability, all sampling procedures were carried out by the same operator for all patients, ensuring consistency in sampling procedure. At the end of sampling procedures, four samples were obtained for each patient: two from the dominant palm and two from the oral cavity. The experimental protocol overview is represented in Figure 1 (35). The mean abundances of microorganisms from the two samples collected at each anatomical site were calculated to generate two stable representative microbiota profiles for each patient’s skin and oral cavity, respectively. These microbiota profiles were considered reflective of the patient’s baseline state prior to the intervention, enhancing the accuracy of subsequent analyses, reducing temporal variability and increasing the robustness of each patient’s composite microbial profile.

Based on patients’ medical history and sampling site, reference microbiota profiles were assigned to four groups: group 1: HAI_Oral (oral samples from the “case” group), group 2: NoHAI_Oral (oral samples from the “control” group), group 3: HAI_Skin (skin samples from the “case” group), and group 4: NoHAI_Skin (skin samples from the “control” group).

Sample collection took place on different days, depending on the surgical period. After each collection, microbial DNA was immediately extracted and stored at −20 °C to preserve its quality and stability. Once all samples were collected and the microbial DNA extracted, subsequent analyses were carried out simultaneously on all the samples.

Patient data, including age, gender, ASA score, and others clinical and surgical information, were collected and stored in a computerized database protected by a password known only to authorized researchers.

### 2.3 Microbial DNA extraction and library preparation

Microbial DNA extraction from oral and skin samples was performed using QIAamp PowerFecal Pro DNA Kits (QIAGEN, Hilden, Germany), following the same procedure described in Delicati et al. (36) Subsequently, library preparation, microbial DNA sequencing, and initial bioinformatics analysis were performed by an external facility (Personal Genomics SRL, Verona, Italy). Hypervariable regions V3-V4 of the 16S rRNA gene were amplified using primers combination Pro341F and Pro805R and sequenced with Illumina MiSeq platform considering 300 paired-end (37).

### 2.4 Clinical and demographic statistical analysis

Clinical and demographic analyses were performed to explore potential confounding variables associated with HAIs, considering both “case” and “control” groups. Variables included patient characteristics (such as ASA score, comorbidities, and chronic therapies), surgical features (season, duration, type, re-intervention, and days of hospitalization), and device parameters (number and length of implanted devices). Univariate analyses were conducted for each of these variables using the T-test or Mann-Whitney U test for continuous variables (e.g., days of hospitalization and duration of surgery) and the Chi-Square test or Fisher’s exact test for categorical variables (e.g., season of surgery, ASA score), depending on data distribution. Results were considered significant when the *p*-value was less than 0.05.

### 2.5 Bioinformatic and statistical analysis of microbiota

After analysing the clinical and demographic characteristics of the patients, microbiological analyses were performed to investigate differences in microbiota profiles between patients who developed HAI and those who did not. Microbiological data were processed and analyzed using Qiime2 (release 2023.9) and R studio (version 4.3.22023.10.31 ucrt) (38–40).

KronaTools was used to generate Krona charts which represent the mean relative abundance of different microorganisms in the four experimental groups (HAI_Oral, NoHAI_Oral HAI_Skin, and NoHAI_Skin) (41). Subsequently, to define the patients’ reference microbiota (for both oral and skin sites), it was necessary to compare the two samples collected one month apart from the same site, as previously mentioned. Relative phylum frequencies were visualized using a stacked bar-graph (38).

Since age and gender balance was considered adequate, statistical analyses were performed treating the groups as independent. Normality was assessed using the Shapiro–Wilk test and parametric (T-test) or non-parametric (Mann–Whitney U test) analyses were chosen accordingly. Comparisons were performed separately for each anatomical site (oral and skin), avoiding to compare all groups together to limit the risk of false positive. These analyses focused on differences at the domain, phylum, and genus levels.

Additionally, differences at the phylum level were evaluated between oral and skin samples to confirm their different biological origins. A preliminary evaluation of outliers, using interquartile range, was conducted. Statistically significant results were visualized using bar charts or heatmap. A *p*-value lower than 0.05 was considered significant.

At the species level, further in-depth analyses were performed focusing on alpha diversity and beta diversity. In particular, alpha diversity was assessed through rarefaction curves, calculated using the Shannon index and Species Richness, at a sequencing depth of 20,000 reads. Instead, beta diversity was evaluated using Principal Coordinate Analysis (PCoA) plot based on Aitchison distance, which accounts for the compositional nature of microbiome data. Eventually, an Indicator Value (IndVal) analysis was performed to identify specific species significantly associated with either the HAI or NoHAI groups in oral and skin sites. Taxa with significant IndVal (*p*-value < 0.05) were considered potential biomarkers. To ensure reproducibility, a random seed of 42 was used in the IndVal analysis during permutation tests to calculate statistical significance.

## 3 Results

In this study, microbiological samples were collected and analyzed from patients undergoing VEGs implantation, with the aim of identifying potential associations between the human microbiota and the onset of HAIs. Firstly, to confirm the reliability of the “non-one-to-one matching” process based on age and gender of the patients, in the “case” and “control” groups, group balance was assessed through univariate analyses. The results showed no statistically significant differences between the groups for age and gender (*p*-value > 0.05), confirming adequate balance for these variables. Subsequently, univariate analyses were performed to assess the impact of other potential confounding variables on the propensity to develop HAIs. None of these variables showed significant results, except for hospitalization days, which had a median of 2.5 days (interquartile range (IQR) of 1.75) in “control” group and of 14.5 days (IQR = 11) in “case” group (*p*-value = 0.002). Regarding the other analyzed variables, which did not differ significantly between groups, the results were as follows (mean ± st. dev. are reported for continuous variables unless otherwise specified): (i) most patients in both groups had an ASA score of 3 (*p*-value = 0.226); (ii) the mean number of comorbidities was 4.40 ± 1.35 in the “control” group and 4.50 ± 1.96 in the “case” group (*p*-value = 0.896); (iii) the mean number of chronic therapies was of 4.80 ± 1.32 in “control” group, whereas in the “case” group it was 4.30 ± 1.49 (*p*-value = 0.438); (iv) in the “case” group, most patients underwent surgery in spring, whereas in the “control” group most operations took place in winter (*p*-value = 0.093); (v) the duration of surgery had a median value of 3.00 h in both groups but with an IQR of 0.75 in the “control” group and of 3.50 in the “case” group (*p*-value = 0.424); (vi) all surgeries in the “control” group were planned, while approximately 40% of patients in the “case” group underwent emergency surgery (*p*-value = 0.087); (vii) one patient per group required a second operation (*p*-value = 1.000); (viii) the mean number of implanted VEGs was 4.10 ± 2.56 in the “control” group and 3.60 ± 2.01 in the “case” group (*p*-value = 0.633); and (ix) the total length of the implanted VEGs was comparable between groups (313.70 ± 137.33 mm in the “control” group and 318.80 ± 199.08 mm in the “case” group; *p*-value = 0.948).

All implanted devices were antimicrobial-free, made of similar materials, and implanted in analogous anatomical sites in both groups. Additionally, all patients received antibiotic prophylaxis with cefazolin both before and after surgery. Data related to the variable described above, for which univariable analyses were performed, are summarized, for each patient, in Supplementary Table 1. As the groups were comparable in terms of clinical variables, age, and gender, subsequent statistical analyses for potential microbiological predictors of HAIs development were conducted treating the groups as independent.

Further descriptive analyses focused on the characteristics of the HAIs that affected the patients in the “case” group (summarized in Supplementary Table 2). HAIs were detected on average 6.6 days after the surgery with a st. dev. of 8.7 days. Six patients developed lung infections; of these, five presented radiological evidence of lung consolidations, suggestive of pneumonia or other respiratory conditions, although no specific pathogens were identified. One patient developed a lung infection caused by the fungus *Candida albicans*. Two patients developed urinary tract infections (UTIs): one due to *Escherichia coli*, and one with no specific pathogens identified. One patient developed a surgical wound infection caused by *Enterobacter cloacae,* and another developed a bloodstream infection caused by *Staphylococcus haemolyticus*.

Subsequently, microbiological analyses were performed to compare the microbiota profile between the HAI and NoHAI groups, revealing differences in both composition and diversity at the oral and skin levels. Specifically, individual oral and skin samples were used to generate two reference microbiota profiles per patient – one for each anatomical site – by averaging the microbial results of the two sampling time points. These profiles were categorized into the four experimental groups: HAI_Oral, NoHAI_Oral, HAI_Skin, and NoHAI_Skin. This classification allowed for the investigation of correlations and differences between the two groups from the oral cavity (HAI_Oral vs. NoHAI_Oral) and the two groups from the skin (HAI_Skin vs. NoHAI_Skin), separately.

**Figure 2:**
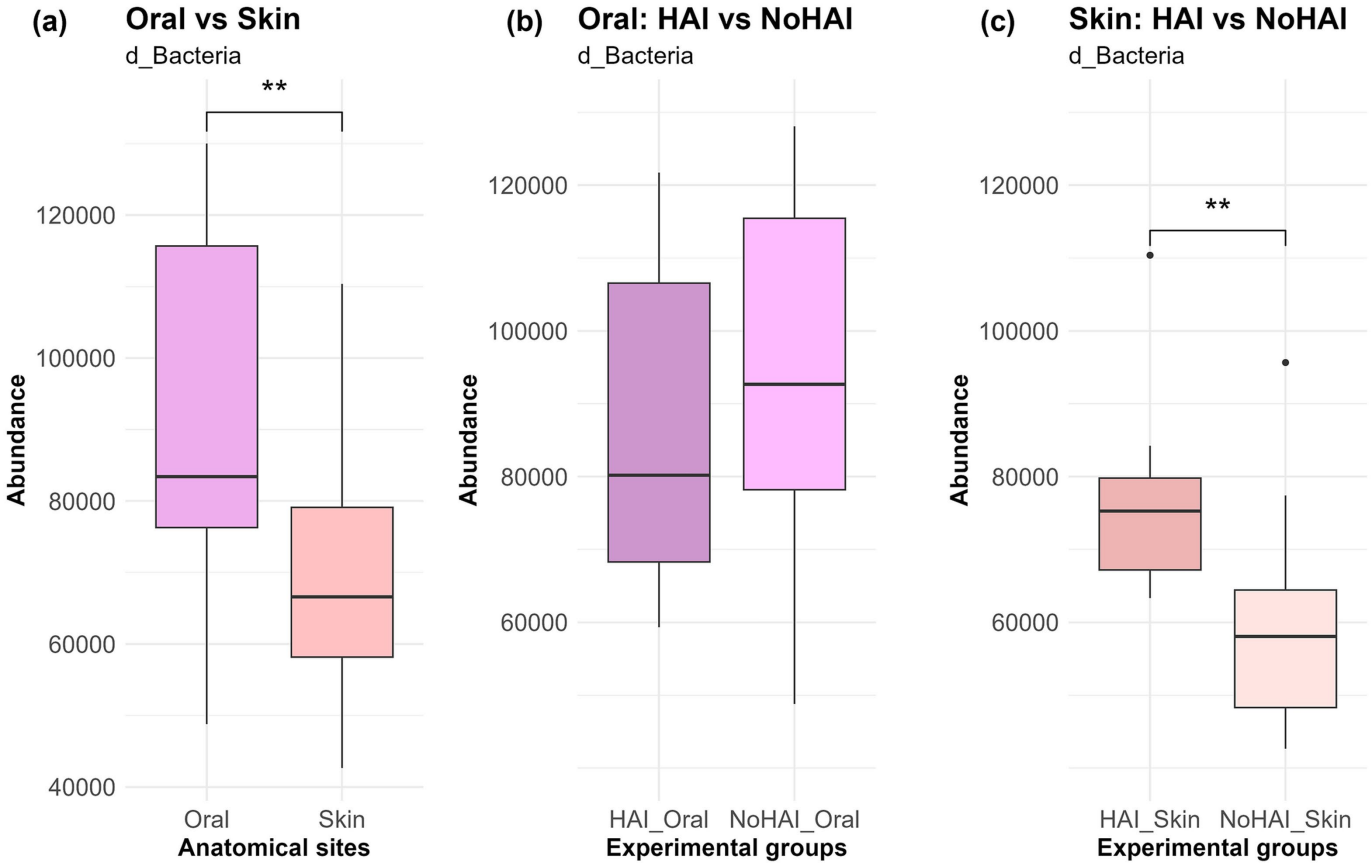
Bacteria domain in oral and skin samples. **(a)** Overall abundance of d_Bacteria among anatomical sites, independent of infection onset. **(b)** Differences in abundance between HAI and NoHAI groups in oral samples. **(c)** Differences in abundance between HAI and NoHAI groups in skin samples. Significance levels are indicated as follows: *p-value < 0.05, **p-value < 0.01, ***p-value < 0.001, and ****p-value < 0.0001. HAI, healthcare-associated infection.

Krona charts, representing the mean relative abundance of microorganisms across the different taxonomic levels for the four experimental groups, were generated (Supplementary Figures 1a–d). Subsequently, further analyses were performed across different taxonomic level (domain, phylum, genus, and species) to deepen the understanding of human microbiota’s correlation with the onset of HAIs. At domain level, the vast majority of the identified microorganisms were classified as bacteria (Supplementary Figures 1a–d), consistent with 16S rRNA sequencing. As displayed in Figure 2, oral samples displayed a higher bacteria abundance in comparison to skin samples (*p*-value < 0.01). However, at oral level, this abundance resulted lower in patients who developed HAIs after the surgery (HAI_Oral) than in those who did not (NoHAI_Oral). In contrast, the skin microbiota showed an increase in the abundance of microbial species associated with the microbiota HAI_Skin than in NoHAI_Skin (Figure 2). Notably, while the trend observed for the oral microbiota did not reach statistical significance, in the skin microbiota the difference between the HAI and NoHAI groups was statistically significant (*p*-value = 0.007).

**Figure 3:**
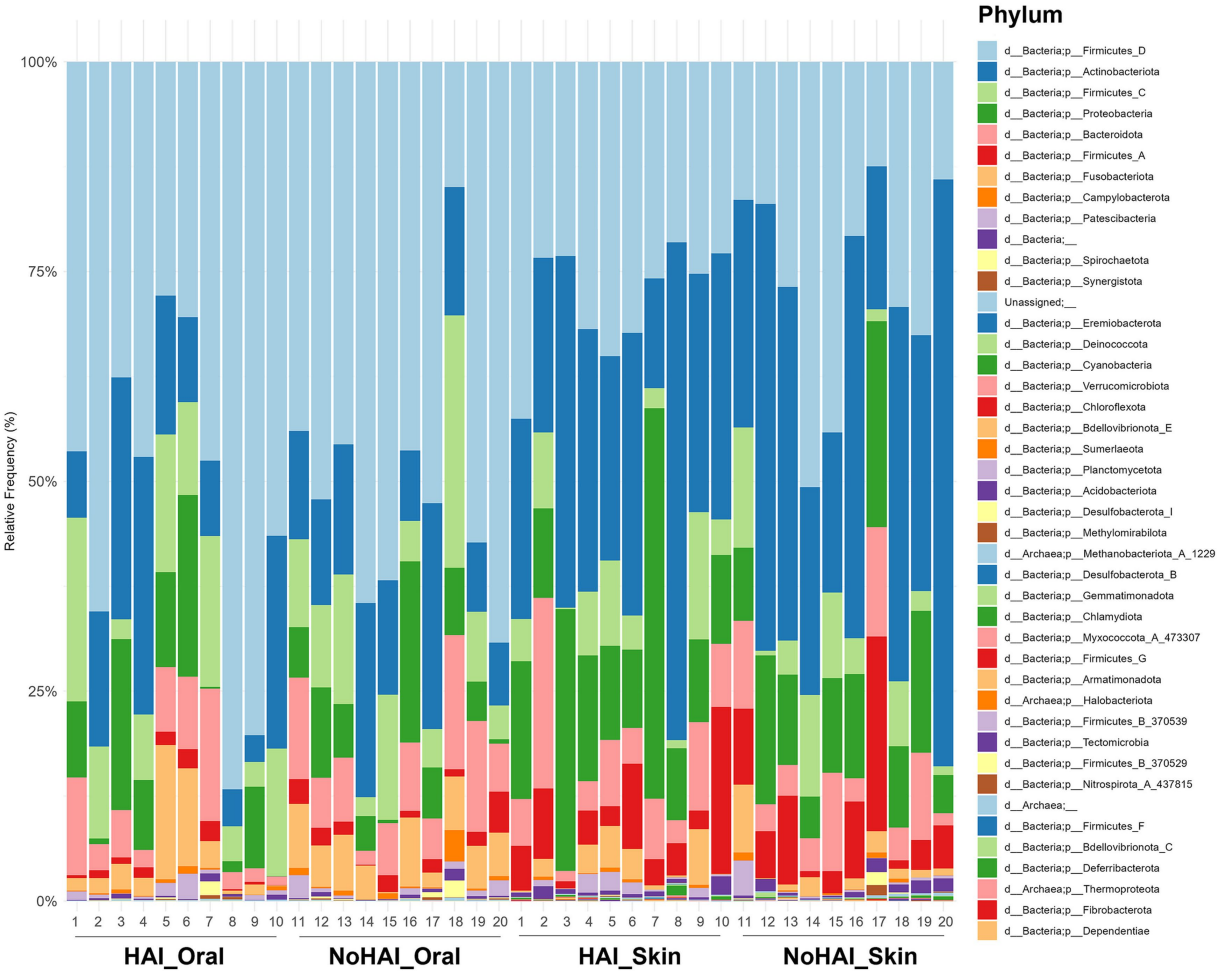
Phylum-level relative frequencies. Stacked bar graph representing the relative frequency of bacteria phylum across the four experimental groups (HAI_Oral, NoHAI_Oral, HAI_Skin, NoHAI_Skin). HAI, healthcare-associated infection.

Phylum-level analyses were conducted to explore the relative abundances of microbial phyla across all samples. A stacked bar graph was generated to visualize the distribution of these phyla in HAI and NoHAI groups at both oral and skin levels (Figure 3). As illustrated, Firmicutes D was significantly more abundant in the oral microbiota with a value of 52% than in the skin microbiota, which displayed a value of 28.5%, showing a statistically significant difference (*p*-value = 0.00002). In contrast, Actinobacteriota was significantly more abundant (*p*-value = 0.002) in the skin microbiota (33.5%) than in the oral microbiota (14.5%). Other phyla showed significant site-specific differences, including Firmicutes C (10.5% oral vs. 6.5% skin; *p*-value = 0.001), Fusobacteriota (5% oral vs. 2% skin; *p*-value = 0.003), and Firmicutes A (1.5% oral vs. 6% skin; *p*-value = 0.0005). Proteobacteria were found to be more abundant on the skin (8% oral vs. 13.5% skin), though this difference was not statistically significant. No other phyla showed statistically significant differences.

**Figure 4:**
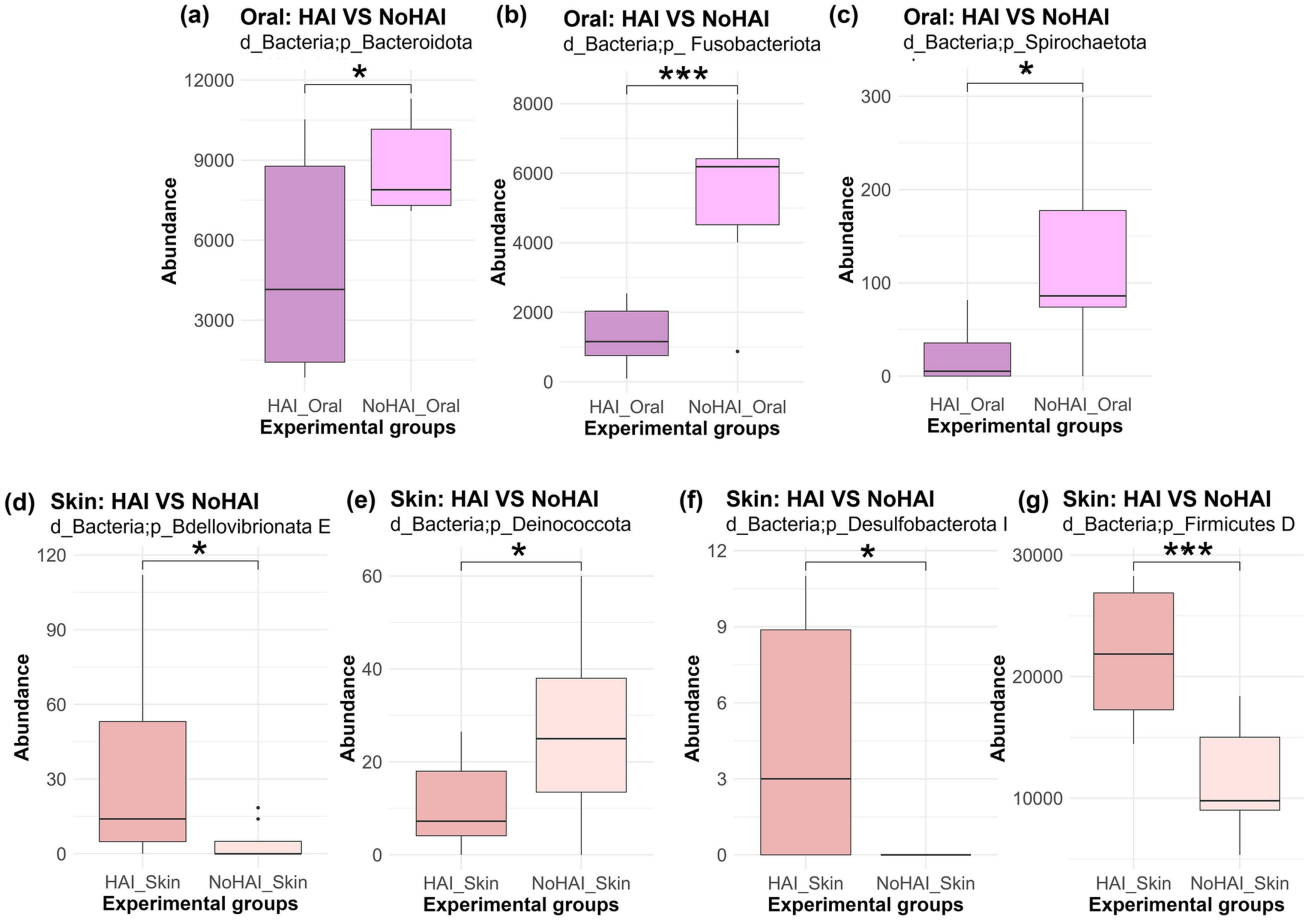
Phylum abundance differences between HAI and NoHAI groups in oral and skin samples. Significant differences among phylum in the different experimental groups are shown according to the anatomical site. The top panels are referred to oral samples: **(a)** Bacteroidota, **(b)** Fusobacteriota, and **(c)** Spirochaetota. The bottom panels are referred to skin samples: **(d)** Bdellovibrionota E, **(e)** Deinococcota, **(f)** Desulfobacterota I, and **(g)** Firmicutes D. The significance levels are indicated as follows: *p-value < 0.05, **p-value < 0.01, ***p-value < 0.001, and ****p-value < 0.0001. HAI, healthcare-associated infection.

Subsequently, phylum-level analyses were further developed by comparing HAI and NoHAI groups separately for oral and skin microbiota. The results, highlighting the significant differences found, are shown in Figure 4. In the oral microbiota, all phyla with significant differences, were more abundant in the NoHAI_Oral group. Among these, Bacteroidota and Spirochaetota showed a moderate significance with *p*-values approximately of 0.02, in both cases, whereas Fusobacteriota showed a highly significant difference (*p*-value < 0.001). Differently, in skin microbiota, only the phylum Deinococcota was significantly higher in NoHAI_Skin group (*p*-value = 0.04), whereas Bdellovibrionota E (*p*-value = 0.049), Desulfobacteriota I (*p*-value = 0.013), and Firmicutes D (*p*-value < 0.001) were more abundant in patients who developed HAIs (HAI_Skin).

**Figure 5:**
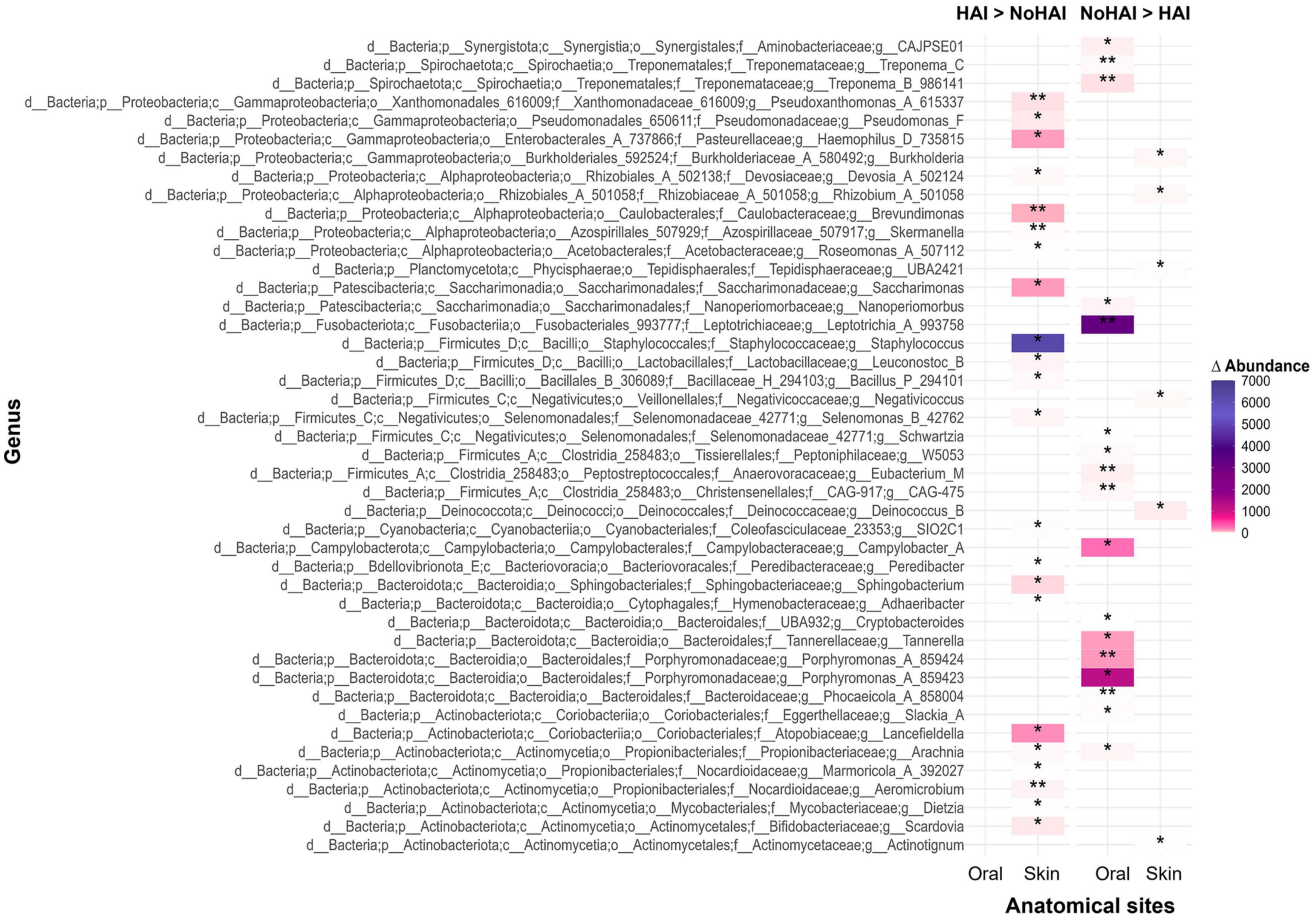
Heatmap of genus-level distribution across experimental groups. The heatmap displays the genera with statistically significant differences in HAI and NoHAI groups, analyzed separately for oral and skin microbiota. HAI, healthcare-associated infection.

Continuing the analysis at the genus level, significant differences emerged between HAI and NoHAI patients. The results, displayed in a heatmap, are reported in terms of absolute differences in median relative abundance between groups, highlighting the genera that were more abundant in patients who developed HAI (HAI > NoHAI) or those that were more abundant in patients who have not developed infections (NoHAI > HAI), as illustrated in Figure 5. In the oral microbiota, no genus was significantly more abundant in HAI_Oral group, whereas several were enriched in NoHAI_Oral group, including *Leptotrichia* (*p*-value = 0.01), *Campylobacter A* (*p*-value = 0.024), *Tannerella* (*p*-value = 0.04), and *Porphyromonas A 859423* (*p*-value = 0.02).

In contrast, in the skin microbiota, most genera that showed significant differences were more abundant in HAI_Skin group. The most abundant genera that showed relevant differences and which, therefore, were considered of particular interest were *Haemophilus D* (*p*-value = 0.036), *Brevundimonas* (*p*-value = 0.008), *Saccharimonas* (*p*-value = 0.022), *Staphylococcus* (*p*-value = 0.011), and *Lancefieldella* (*p*-value = 0.027). Only a few genera resulted more abundant in patients of the NoHAI_Skin group, although the median differences were minimal.

**Figure 6:**
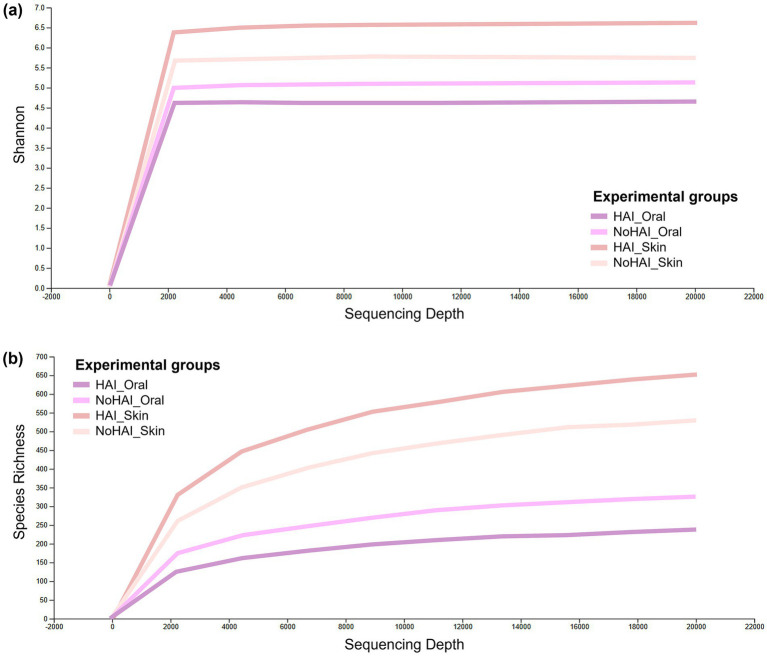
Alpha rarefaction curves. Graphical representation of alpha rarefaction curves across experimental groups based on **(a)** Shannon index and **(b)** Species richness.

The final analyses were focused on the last taxonomic level, those of the species. Rarefaction curves based on Shannon indexes and Species Richness reached a stable plateau around 2,000 reads for all groups, indicating that a sufficient level of species exhaustion was reached at this sequencing depth (Figure 6). Although all curves reached a similar plateau, the Shannon diversity and Species Richness values varied between groups, suggesting differences in microbial compositions. In particular, the HAI_Skin group showed the highest Shannon diversity value, with a value of around 6.5, significantly higher than the NoHAI_Skin group (mean ≈ 5.5; *p*-value = 0.023, Kruskal-Wallis test). In contrast, the HAI_Oral group showed a lower Shannon diversity (mean ≈ 4.5) than the NoHAI_Oral group (mean ≈ 4.8), but this difference was not statistically significant (*p*-value = 0.597). These findings indicate a potential site-specific dysbiosis pattern, with increased skin microbiota diversity and decreased oral microbiota diversity in patients who developed HAIs (Figure 6a).

The order of the groups in terms of Species Richness is essentially the same, but it was observed that the plateau is reached more gradually, suggesting a different species composition or density between the groups. In particular, the skin groups, both HAI and NoHAI, showed higher species abundance than the oral groups, reflecting the different microbial ecologies of the two anatomical sites (Figure 6b).

**Figure 7:**
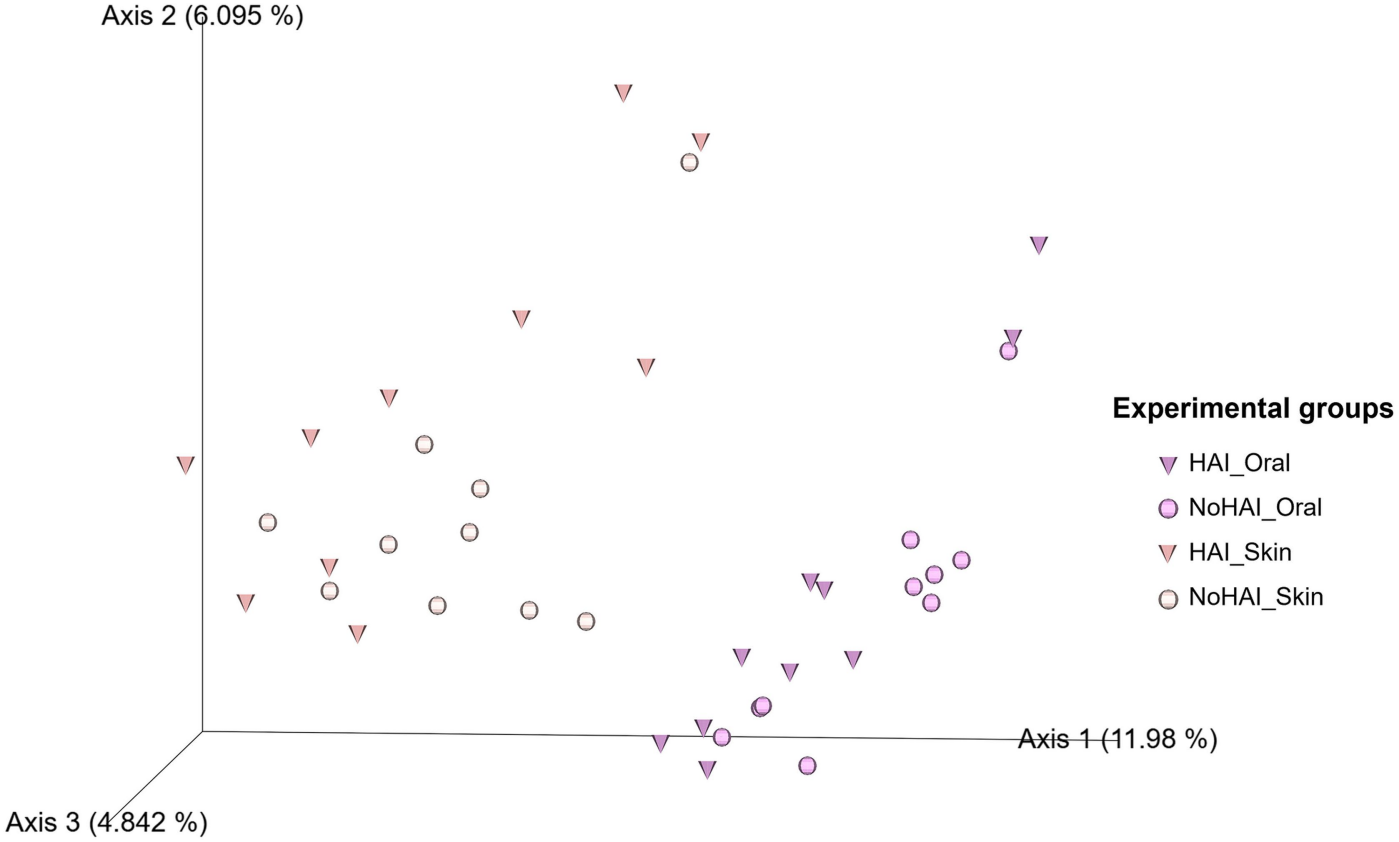
Principal coordinate analysis (PCoA) plot of beta diversity based on Aitchison distance. In the 3D PCoA plot the beta diversity distribution among samples is visually represented. Triangles represent samples from patients who developed HAI after the vascular endografts (VEGs) implantation, whereas the circles represent those from patients who did not. HAI, healthcare-associated infection.

Beta diversity assessed by Aitchison distance and represented via a PCoA plot (Figure 7) showed a clear separation between oral and skin samples along Axis 1, which explains 11.98% of the total variation. Notably, both HAI_Oral and HAI_Skin groups displayed greater dispersion along Axis 2 (explaining about 6.10% of the total variation) and Axis 3 (accounting for about 4.84%), in particular in HAI_Skin samples.

**Figure 8:**
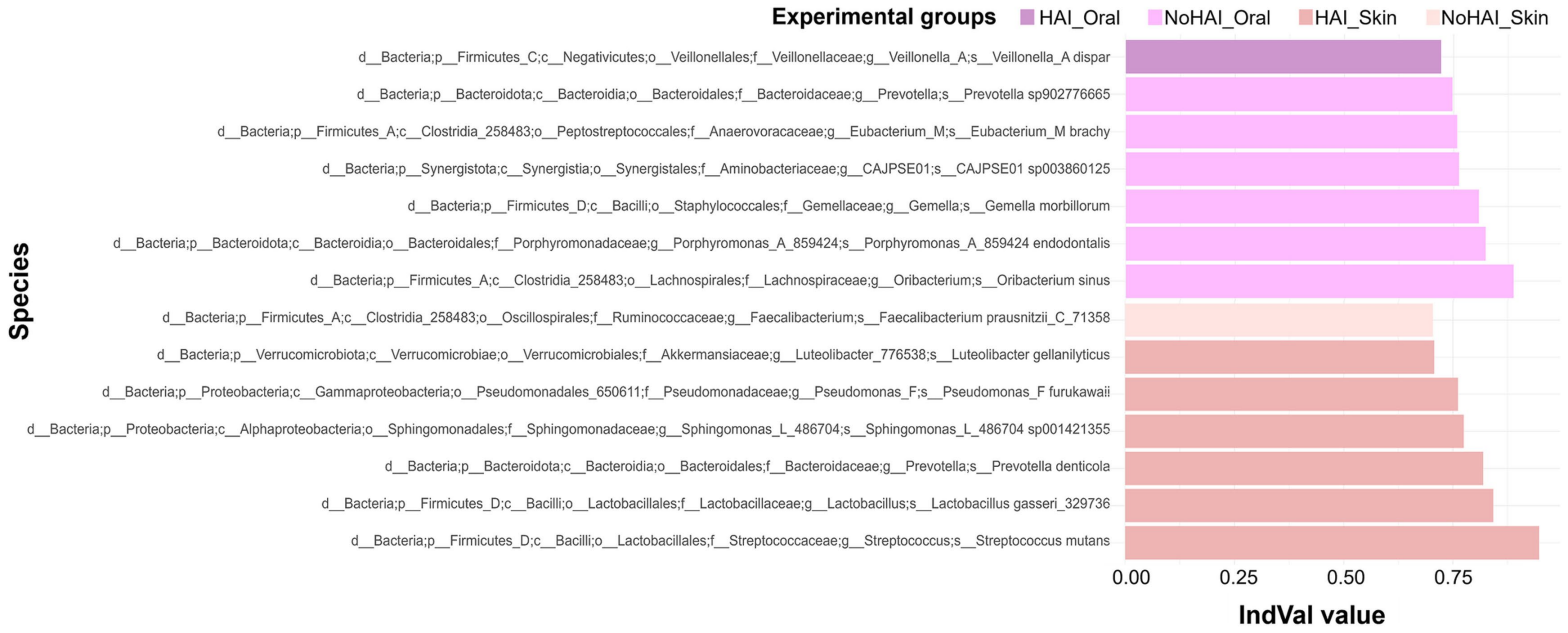
Indicator Value (IndVal) analysis for oral and skin microbiota. Analysis of the species which characterized the different groups based on their specificity and fidelity across samples. The Y axis list the taxonomic designation of microbial species, whereas the X axis the corresponding IndVal value.

Finally, IndVal analysis allowed to identify potential bacterial species significantly associated with the HAI and NoHAI groups in oral and skin samples. The species identified in each group reflect significant differences in microbial composition and may provide clues about the predisposition to develop HAIs. Among more than 2,000 species analyzed, 14 species have been highlighted as significant in characterizing the experimental groups (Figure 8). In particular, in oral samples, *Veillonella A. dispar* was the only species significantly associated with the HAI_Oral group with an IndVal value of approximately 0.72 (*p*-value = 0.035) and six species were significantly more abundant in the NoHAI_Oral group, with IndVal values ranging from 0.74 to 0.88: *Prevotella sp902776665* (IndVal = 0.74, *p*-value = 0.025), *Eubacterium M brachy* (IndVal = 0.75, *p*-value = 0.03), *CAJPSE01 sp003860125* (IndVal = 0.76, *p*-value = 0.035), *Gemella morbillorum* (IndVal = 0.80, *p*-value = 0.025), *Porphyromonas A 859424 endotalis* (IndVal = 0.82, *p*-value = 0.015), *Oribacterium sinus* (IndVal = 0.88, *p*-value = 0.035). On the other hand, considering skin samples, six species were significantly more abundant in the HAI_Skin group with IndVal values ranging from 0.71 to 0.94: *Luteolibacter gellanilyticus* (IndVal = 0.71, *p*-value = 0.03), *Pseudomonas F furukawaii* (IndVal = 0.76, *p*-value = 0.03), *Sphingomonas L 486704* sp. *001421355* (IndVal = 0.77, *p*-value = 0.01), *Prevotella denticola* (IndVal = 0.82, *p*-value = 0.01), *Lactobacillus gasseri 329,735* (IndVal = 0.84, *p*-value = 0.045), and *Streptococcus mutants* (IndVal = 0.94, *p*-value = 0.005). Moreover, only one species, *Faecalibacterium prausnitzii* (IndVal = 0.70, *p*-value = 0.045), was significantly associated with the NoHAI_Skin group.

## 4 Discussion

This study focused on exploring potential associations between the human microbiota and the occurrence of HAIs in patients undergoing VEGs implantation. Investigating both the skin and oral microbiota allowed consideration of microbial communities across different body sites in the context of post-operative HAIs, providing a broader perspective on potential risk factors. In fact, the occurrence of HAI in patients who underwent abdominal graft implantation could lead to VEGs infections (42), an uncommon yet life-threatening condition that requires a combination of medical and surgical treatment, the latter consisting in eradication of the infected graft, followed by in-situ reconstruction with biological or prosthetic material, a procedure associated with high rates of morbidity and mortality (43).

Microbiological samples were collected 12 months after surgery, with a second collection after one month, to capture relatively stable microbiota, minimizing the influence of transient factors such as seasonal fluctuations, drug therapies or dietary variations. Two reference microbial profiles per patient (oral and skin) were generated, increasing data reliability.

Another strength of the study is represented by the selection of patients. The comparison groups were balanced for age and gender, ensuring basic homogeneity between the cohorts. This minimized demographic bias, increasing the reliability of the analyses. Preliminary checks confirmed no significant differences in age or gender between groups allowing us to focus on microbiota-related differences while considering the groups as independent.

A total of 20 patients, categorized as “case” and “control” groups were selected and information about demographics, surgery, hospitalization, and devices were collected (Supplementary Table 1). Although literature suggests that ASA score, comorbidities, and chronic therapies may influence HAIs risk (44–49), none of these variables showed significant differences between groups in our cohort, suggesting relative homogeneity. The only exception was the length of hospitalization (*p*-value = 0.002). However, this result is expected, as longer hospitalization is a direct consequence of the occurrence of HAIs rather than a causal factor, consistent with previous reports (49–52).

HAIs were detected on average 6/7 days post-surgery (Supplementary Table 2), indicating that most infections occurred early, consistent with previous studies (11, 17, 53, 54). The types of HAIs observed in our cohort were heterogeneous (Supplementary Table 2), with a predominance of those related to the lungs, which affected a total of six patients. This higher incidence aligns with other literature data, which reported that the majority of HAIs are associated with lung infections (10, 55). Of these, five patients showed lung consolidations, a clinical condition that may be associated with pneumonia or other respiratory diseases. Interestingly, no specific pathogens were identified in these cases, so these lung consolidations may have been caused by non-infectious factors or by pathogens not detected by the methods used. This is in accordance with the findings of other studies in which a significant proportion of pneumonia cases remain culture-negative (56–58). One patient developed a lung infection caused by *Candida albicans*, a fungal pathogen that is relatively rare in lung infections but is known to cause pneumonia in immunocompromised patients or those undergoing invasive procedures, as seen in our case (59–63). Two patients developed UTIs, one caused by *Escherichia coli* [one of the pathogens most commonly involved in UTIs (64)] and the other with no specific pathogen identified. UTIs are also common among hospitalized patients and are often related to the use of urinary catheters or manipulation of the urinary tract during surgery (65–68). Failure in detecting the causative pathogen of the UTIs can occur sometimes (as occurred for our previously cited patient), especially with multidrug-resistant organisms that may not be easily identified with standard diagnostic methods. However, in the last year new technological approaches are being developed to overcome this problem (69–71). Additionally, as noted, one patient developed a surgical wound infection caused by *Enterobacter cloacae*, a bacterium commonly associated with surgical site infections (72–75). This microorganism is part of the normal flora of the gastrointestinal tract but can become pathogenic in immunosuppressed or postoperative patients (76–78). Finally, a bloodstream infection was detected in one patient, caused by *Staphylococcus haemolyticus*, a species known to cause infections in immunosuppressed patients. This pathogen is part of the normal skin flora but can become invasive, particularly in patients with indwelling devices or undergoing major surgery (79).

These findings collectively highlighted the diverse spectrum of infections that can arise after VEGs implantation and underscore the importance of the human microbiota in the infectious context. Therefore, to explore potential microbiota-related patterns, analyses were performed at different taxonomic levels, from domain and phylum to genus and species, across the two anatomical sites.

At domain level (Figure 2), oral microbiota showed a higher absolute bacteria abundance than skin, consistent with the humid, warm, and nutrient-dense environment of the oral cavity (80, 81). Conversely, the lower bacteria abundance on skin reflects its desiccated, nutrient-poor, and exposed conditions (82). Although the oral microbiota is generally characterized by a higher bacterial content compared to the skin microbiota, we observed that microbial diversity differed between HAI and NoHAI patients. Specifically, Shannon diversity decreased in oral samples (*p*-value = 0.597) and increased in skin samples (*p*-value = 0.023) among HAI patients compared to controls, indicating a significant difference only for the skin microbiota (Figure 6). This result confirms other data recently published by Wang et al. (83) and Ogbanga et al. (84), possibly reflecting the greater temporal variability of the skin microbiome (85, 86).

Phylum-level analyses confirmed expected differences between skin and oral microbiota (Figure 3 and Supplementary Figures 1a–d). The results of the phylum-level analyses confirmed the known differences in the composition of the oral and skin microbiota, highlighting how the microbial diversity and the relative abundance of the main phyla are closely linked to the anatomical environment of origin (87). For example, Firmicutes D was more abundant in the oral cavity (52% vs. 28.5%, *p*-value = 0.00002), while Actinobacteriota shows a greater abundance in the skin (33.5% vs. 14.5%, *p*-value = 0.002). These differences align with known functional and environmental characteristics of the sites (88–91). Other phyla, such as Firmicutes C, Fusobacteriota, and Firmicutes A, also showed similar trends, highlighting the compositional differences between the skin and oral microbiota, whereas Proteobacteria were similar between sites as observed in other studies (83, 92–97). Overall, these results confirm the well-known distinction between the microbial composition of the oral and skin microbiota at the phylum level.

Comparing HAI and NoHAI patients, oral samples showed a non-significant reduction in bacterial abundance, interpretable as descriptive trend, in HAI_Oral compared to NoHAI_Oral groups, whereas skin samples showed a significant increase (*p*-value = 0.007) in bacteria abundance in HAI_Skin vs. NoHAI_Skin (Figure 2). These results are in line with those obtained from the analysis of microbial diversity alpha rarefaction curves, previously described. In particular, the reduction in oral microbial diversity in HAI patients may indicate a dysbiotic state, potentially predisposing to opportunistic colonization, although causality cannot be established (98–101). Conversely, the increase in skin microbial diversity in HAI patients seem to be in contrast with some studies suggesting a reduced skin microbial diversity as a consequence of infections, skin diseases, or antibiotic exposure (102, 103). However, our observation can be explained in the light of a more complex phenomenon, which considers different contexts and phases of the disease. Although, this increase does not imply a direct pathogenic role; it may reflect an increase in colonization by opportunistic or environmental taxa under certain clinical conditions (104, 105).

To further explore variations in microbial composition between experimental groups, beta diversity was assessed using a PCoA plot based on the Aitchison distance, which assesses the overall dissimilarity between microbial communities. The PCoA plot (Figure 7) revealed clear distinctions by anatomical site along Axis 1 (11.98% of total variation), consistent with previous findings suggesting a substantial difference in microbial composition between oral and skin microbiota (84, 87). Within each anatomical site, HAI samples showed greater dispersion along Axis 2 and Axis 3 (6.10 and 4.84% of the total variation), suggesting increased heterogeneity in microbial communities of patients who developed infections. This is in line with the notion that infections can be the result of different significant changes in the human microbiota, but causal links to HAI development cannot be yet established (106). Notably, skin samples from the HAI group (HAI_Skin) showed the most pronounced variability. Overall, these results indicate differences in microbial structure between patients and anatomical sites, but findings remain exploratory.

Subsequently, analyses at phylum, genus, and species levels identified taxa that distinctly characterize HAI and NoHAI groups. At phylum-level, some phyla were identified with significantly different abundances among experimental groups (Figure 4). Bacteroidota, Fusobacteriota, and Spirochaetota were most abundant in the NoHAI_Oral group, suggesting their potential protective or neutral role. This result is consistent with literature studies that attribute to these phyla a central role in maintaining a balanced oral microbiota and in the regulation of local immunological processes (92, 107–111). For skin, Deinococcota was more abundant in NoHAI samples, potentially reflecting a stable microbiota (112). On the contrary, Bdellovibrionota E, Desulfobacterota I, and Firmicutes D were detected as most abundant in HAI_Skin group, these differences may indicate altered microbiota composition, however functional and predictive implications should be further explored (107, 113–117).

At genus level the results highlighted the importance of specific microbial genera in differentiating between patients who developed HAIs and who did not (Figure 5). In the oral microbiota, several commensal oral genera (e.g., *Leptotrichia, Campylobacter A*, *Tannerella*, and *Porphyromonas*) were more abundant in NoHAI group (81, 118–122). On the contrary, an increased abundance of specific genera (including *Staphylococcus, Haemophilus D, Brevundimonas, Saccharimonas, and Lancefieldella*) was detected in patient who developed HAIs in skin samples. While some of these genera include opportunistic pathogens, our preliminary findings can only suggest a possible, but not definitive, association with HAI development. Their increased abundance may rather reflect microbial imbalance or increased susceptibility to infections, emphasizing the need for targeted studies to clarify their functional roles (123–135).

In addition to the most abundant taxa, less represented bacterial genera also emerged as potentially related to NoHAI or HAI groups. Although present in lower amount, such minor taxa may still play a crucial role in host–microbe interactions and dysbiotic process (136, 137). For instance, in our case, *Skermanella* was identified as significantly enriched in the HAI_Skin group. This genus, typically of environmental origin, may indicate exogenous contaminations or alterations in the skin barrier integrity (138–140).

The IndVal analysis was performed at the species level to identify more specific taxa associated with the HAI and NoHAI groups, in line with previous studies that applied this approach for biomarker discovery (Figure 8) (141, 145). Species associated with NoHAI_Oral (e.g., *Prevotella, Porphyromonas, CAJPSE01 sp003860125, Gemella morbillorum,* and *Oribacterium*) are commonly part of balanced oral microbiota, and may reflect microbial homeostasis rather than direct protection (81, 121, 146–149). The only exception is, *Eubacterium M. brachy*, which, although characterizing the NoHAI_Oral group, has been repeatedly associated with oral diseases and, more rarely, with systemic inflammation (150–152). However, its presence is more likely influenced by host-related factors, such as oral exposure to cigarette smoke, rather than representing a biomarker of individual health (153). Conversely, in HAI_Oral group, *Veillonella A. dispar* was identified (IndVal value ≈ 0.72); while usually commensal, it can contribute to biofilm formation, favouring colonization by opportunistic pathogens. Although the present study did not include a specific assessment of biofilm formation, this finding may suggest a potential microbial interaction mechanism worth exploring in future research (154–157). Moving to species related to skin microbiota, *Faecalibacterium prausnitzii*, a gut commensal known for its anti-inflammatory properties, was associated the NoHAI_Skin group (158, 159). Although, its occasional detection on the skin in our study should be interpreted with caution, previous studies have suggested a potential beneficial role at skin level (160–165). Differently, several bacterial species such as *Luteolibacter gellanilyticus, Pseudomonas F furukawaii, Sphingomonas L 486704 sp001451355, Lactobacillus gasseri, Prevotella denticola* and *Streptococcus mutans* were linked to the HAI_Skin group. These bacteria species are mainly environmental or opportunistic organisms, and their detection on the skin may reflect altered microbial dynamics or transient colonization, potentially predisposing to infections (166–178).

Overall, these results highlight the differences in diversity and composition between HAI and NoHAI groups at the different anatomical sites, but the findings remain exploratory and future studies are required to evaluate whether these microbial patterns could serve as biomarkers for patient risk stratification or targeted preventive interventions. From a translational perspective, understanding these microbial signatures could provide a useful framework for future clinical applications. In particular, microbiota signatures might support the identification of patients at higher risk of developing HAIs before surgery, leading to preoperative risk stratification and to the design of personalized preventive or hygiene interventions aimed at reducing HAIs incidence. Moreover, if validated in larger and longitudinal studies, such biomarkers could complement existing infection control measures, offering a more individualized approach to patient management. Nevertheless, the present data should be interpreted with caution, as clinical translation will require further methodological standardization and independent validation in broader surgical populations.

Indeed, although this study provides preliminary insights into the identification of potential predictive biomarkers associated with susceptibility to HAIs, several limitations must be considered. The study was restricted to patients undergoing VEGs implantation and included a relatively small sample size, limiting the generalizability of the findings. Sample collection was performed 12 months after surgery, with a second sampling one month later. As described above, this timing was intentionally selected to capture a stabilized microbiota and minimizing transient post-surgical influences. Evidence supports that microbial communities tend to re-establish a composition approximating the pre-surgery baseline state, while also minimizing seasonal and daily variability. However, this approach does not capture the microbial state immediately prior to surgery, introducing some uncertainty in linking observed microbiota profiles to HAIs risk. Eventually, additional limitations include the absence of longer-term longitudinal data, potential batch effects from external sequencing, and partially unmeasured patient-related factors such as lifestyle habits (diet, hygiene, sunlight exposure, smoking, etc.) that may act as confounders.

Given these limits, this study should be considered a pilot study. Further targeted investigations are needed to better clarify the specific role of the different taxa as potential predictive biomarker for HAIs. Future work should aim to confirm the findings in a larger cohorts, ideally including patients from broader surgical populations beyond VEGs procedures. Collecting additional information on other patient-related factors will help account for potential confounding variables. Moreover, functional studies – also focusing on less abundant taxa – are necessary to analyse microbial interactions and ecological dynamics which may influence HAIs susceptibility. Finally, incorporating pre-operative sampling could provide a more accurate characterization of baseline microbiota, allowing a clearer understanding of microbial predisposition to HAIs.

## 5 Conclusion

This study provides preliminary evidence of differences in microbiota profiles potentially related to susceptibility to HAIs. Differences in microbial diversity were observed between experimental groups, with lower diversity in the HAI_Oral group and higher diversity in the HAI_Skin group, suggesting that the relationship between microbial diversity and infection risk may be anatomical site-specific. Taxonomic analyses indicated that oral microbiota was more characteristic of the “control” group, while skin microbiota showed features more associated with the “case” group. The taxa identified in the “case” group were often environmental or potentially predisposing to dysbiosis, whereas those in the “control” group appeared more balanced and potentially protective. While these findings highlight microbial profiles that could serve as exploratory biomarkers for HAI susceptibility, the study’s limitations (including small sample size, procedure-specific cohort, post-operative sampling, and unmeasured patient factors) mean that results should be interpreted without generalization. Further research, on larger and more diverse populations, ideally including pre-operative sampling and functional analyses, are needed to validate these observations. Overall, this pilot study underscores the potential relevance of the human microbiota in understanding HAI risk and may inform future approaches for personalized infection prevention with the aim to improve the understanding of HAIs, offering concrete tools to reduce their impact on public health and the healthcare system.

## Data Availability

The datasets generated for this study derive from samples collected from individual participants. In accordance with the approval of the institutional ethics committee and considering the ethical and privacy aspects associated with the nature of the samples, only aggregated data will be made publicly available. Raw sequencing data at the level of individual samples are not deposited in public repositories, but may be provided by the authors to qualified researchers upon reasonable request.
