# Supplementary material for: Microbiota-based biomarkers of infection risk in patients undergoing vascular endograft implantation: a pilot study
Source: Front Med (Lausanne). 2025 Dec 10;12:1680148. doi: 10.3389/fmed.2025.1680148 (PMC12741270; doi:10.3389/fmed.2025.1680148)
Supplement: Supplementary file 1 [file Data_Sheet_1.docx]

Supplementary Material

# Supplementary Tables

*Table 1.* Biological and clinical characteristics of enrolled patients. This table summarized information about patients, surgery, hospitalization, and implanted devices considering the differentiation in “case” (HAI) and “control” (NoHAI) groups. HAI, healthcare-associated infection; VEGs, vascular endografts.

|  | **Patients ID** | | **Age** | **Gender** | **ASA score** | **N° comorbidities** | **N° chronic therapies** | **Season of the surgery** | **Duration of surgery** (hours) | **Type of surgery** | **Re-intervention** | **Hospitalization** (days) | **N° implanted VEGs** | **VEGs total length** (mm) |
| --- | --- | --- | --- | --- | --- | --- | --- | --- | --- | --- | --- | --- | --- | --- |
| Case Group | | **1** | 71 | Male | 3 | 3 | 5 | Autumn | 1 | Emergency | No | 43 | 2 | 70 |
|  |  | **2** | 80 | Male | 3 | 1 | 2 | Winter | 8 | Planned | No | 7 | 6 | 650 |
|  |  | **3** | 77 | Male | 3 | 4 | 3 | Spring | 2 | Planned | No | 2 | 4 | 288 |
|  |  | **4** | 66 | Male | 3 | 4 | 7 | Spring | 6 | Emergency | No | 10 | 2 | 394 |
|  |  | **5** | 88 | Male | 4 | 5 | 5 | Spring | 3 | Emergency | No | 14 | 6 | 495 |
|  |  | **6** | 72 | Male | 4 | 3 | 6 | Spring | 5 | Planned | No | 20 | 1 | 29 |
|  |  | **7** | 76 | Female | 3 | 8 | 3 | Spring | 3 | Planned | No | 15 | 4 | 192 |
|  |  | **8** | 86 | Male | 3 | 6 | 4 | Spring | 2 | Emergency | No | 15 | 4 | 379 |
|  |  | **9** | 83 | Male | 3 | 5 | 4 | Winter | 3 | Planned | No | 5 | 6 | 491 |
|  |  | **10** | 77 | Female | 3 | 6 | 4 | Autumn | 6 | Planned | Yes | 30 | 1 | 200 |
| Control Group | | **11** | 78 | Male | 3 | 6 | 5 | Spring | 3 | Planned | No | 5 | 3 | 380 |
|  |  | **12** | 75 | Female | 3 | 3 | 3 | Autumn | 3 | Planned | No | 3 | 1 | 150 |
|  |  | **13** | 76 | Male | 3 | 4 | 6 | Autumn | 3 | Planned | No | 2 | 5 | 312 |
|  |  | **14** | 84 | Female | 3 | 6 | 4 | Winter | 3 | Planned | No | 1 | 8 | 596 |
|  |  | **15** | 78 | Male | 3 | 5 | 4 | Winter | 2 | Planned | No | 4 | 3 | 309 |
|  |  | **16** | 82 | Male | 3 | 3 | 7 | Winter | 3 | Planned | No | 2 | 6 | 377 |
|  |  | **17** | 82 | Male | 2 | 3 | 3 | Winter | 2 | Planned | No | 2 | 3 | 351 |
|  |  | **18** | 80 | Male | 3 | 6 | 6 | Winter | 3 | Planned | Yes | 1 | 1 | 95 |
|  |  | **19** | 84 | Male | 2 | 3 | 5 | Winter | 2 | Planned | No | 3 | 3 | 240 |
|  |  | **20** | 64 | Male | 3 | 5 | 5 | Autumn | 5 | Planned | No | 9 | 8 | 327 |

*Table 2.* Information on HAIs. This table provides detailed information on the HAIs that affected the “case” group after VEGs implantation. HAI, healthcare-associated infection; VEGs, vascular endografts.

|  | **Patient ID** | **Days between surgery and HAIs detection** | **Type of HAIs** | **HAIs-related pathogen** |
| --- | --- | --- | --- | --- |
| **Case Group** | **1** | 15 | Surgical wound infection | *Enterobacter cloacae* |
|  | **2** | 1 | Lung consolidation | *Not identified* |
|  | **3** | 27 | Urinary tract infection | *Not identified* |
|  | **4** | 0 | Lung consolidation | *Not identified* |
|  | **5** | 1 | Lung consolidation | *Not identified* |
|  | **6** | 1 | Lung infection | *Candida albicans* |
|  | **7** | 10 | Urinary tract infection | *Escherichia coli* |
|  | **8** | 4 | Lung consolidation | *Not identified* |
|  | **9** | 1 | Lung consolidation | *Not identified* |
|  | **10** | 6 | Bloodstream infection | *Staphylococcus haemolyticus* |

# Supplementary Figures


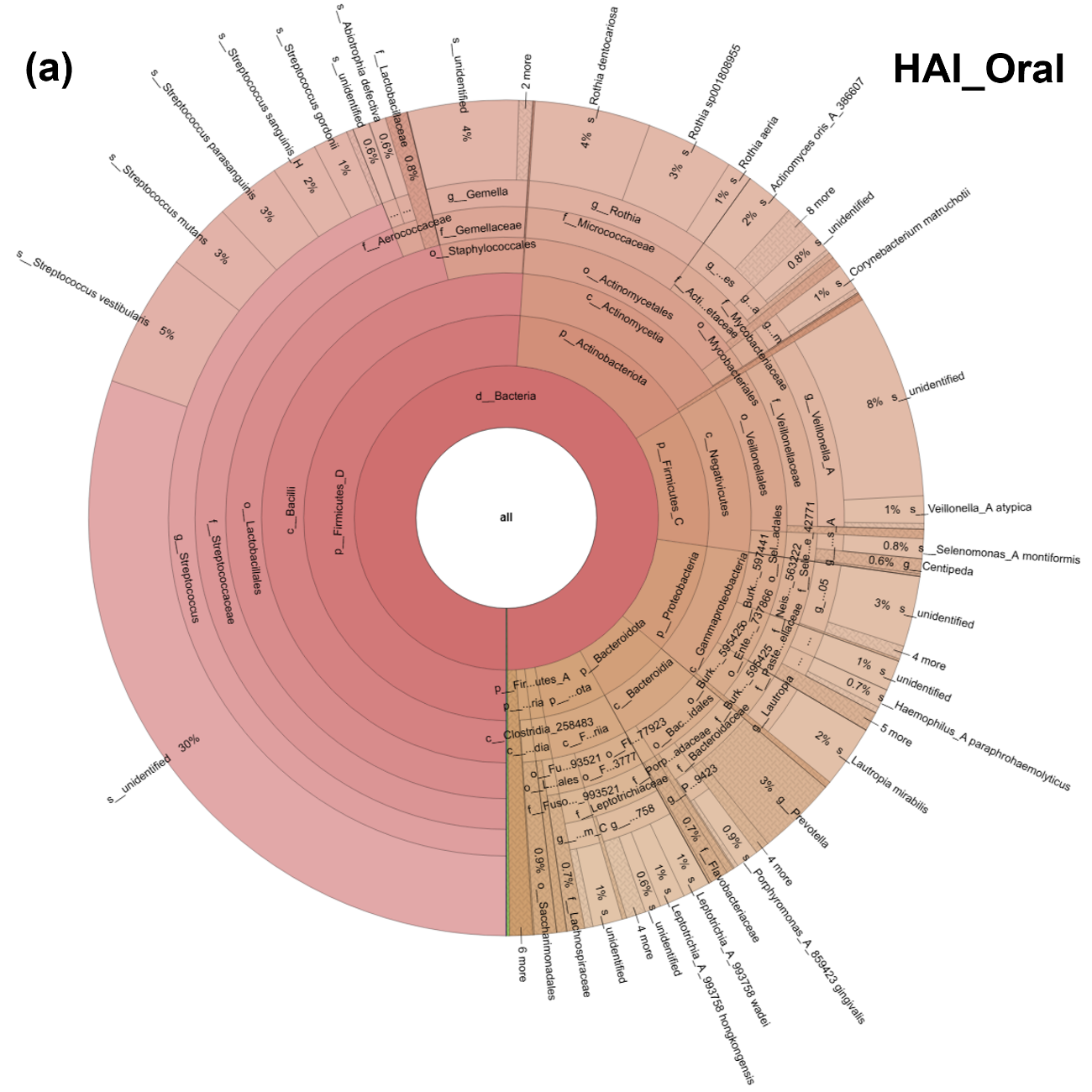


***Figure 1. (a)*** Krona chart representing the average relative bacterial composition in oral samples from patients who developed an HAI after VEGs implantation (HAI_Oral group). HAI, healthcare-associated infection; VEGs, vascular endografts.


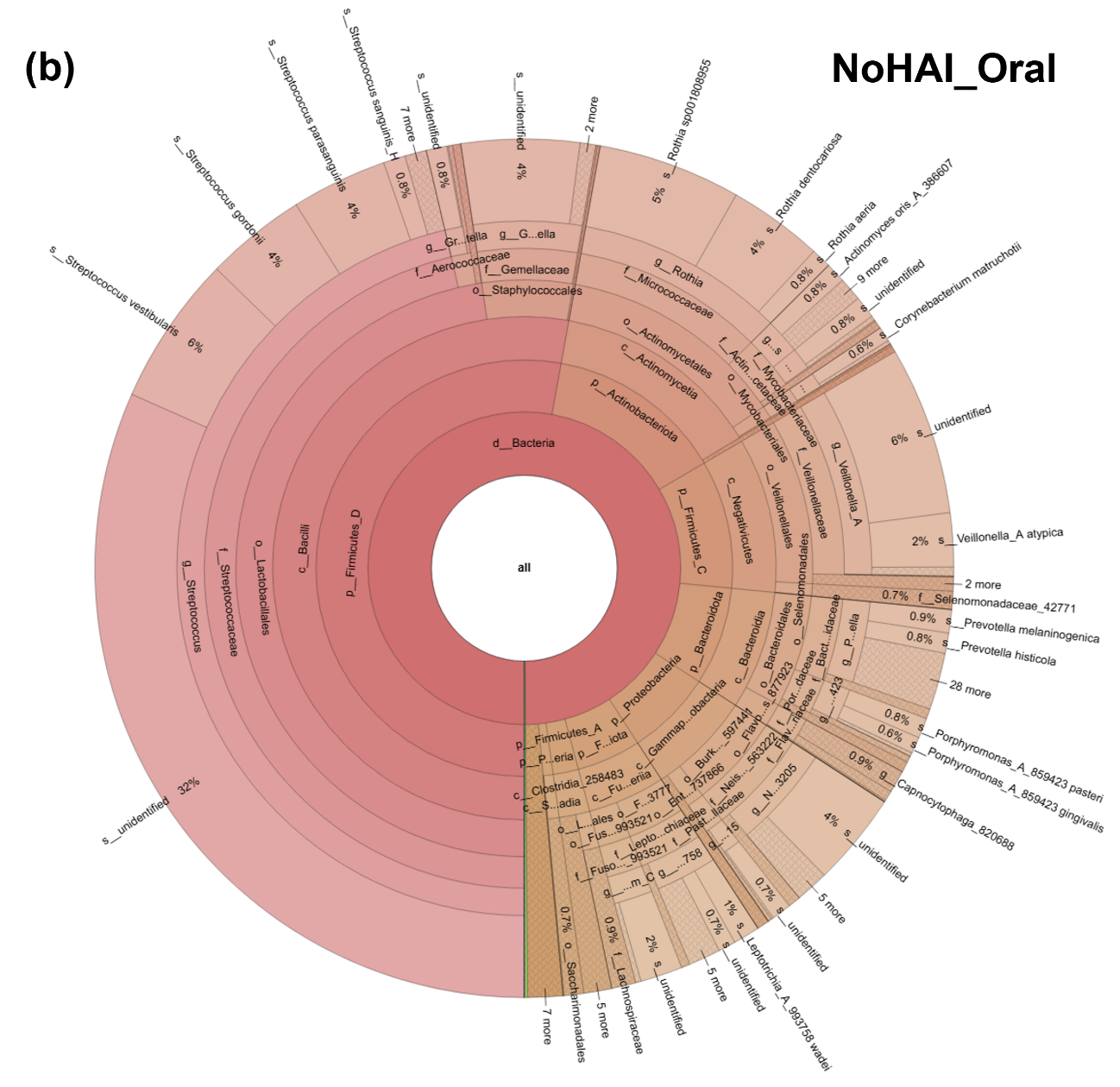


***Figure 1. (b)*** Krona chart representing the average relative bacterial composition in oral samples from patients who did not develop an HAI after VEGs implantation (NoHAI_Oral group). HAI**,** healthcare-associated infection; VEGs**,** vascular endografts**.**


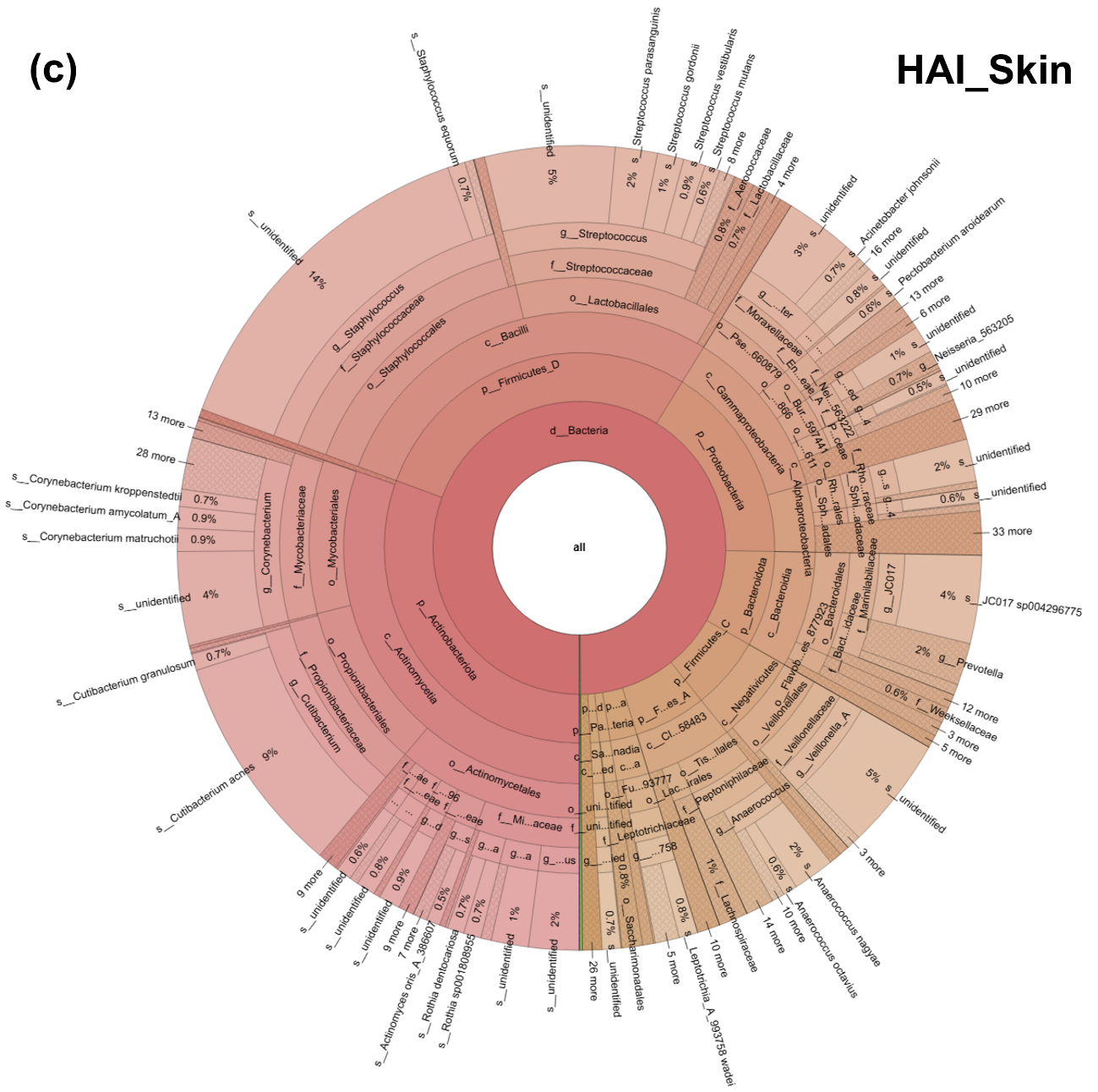


***Figure 1. (c)*** Krona chart representing the average relative bacterial composition in skin samples from the patients who developed an HAI after VEGs implantation (HAI_Skin group). HAI**,** healthcare-associated infection; VEGs**,** vascular endografts**.**


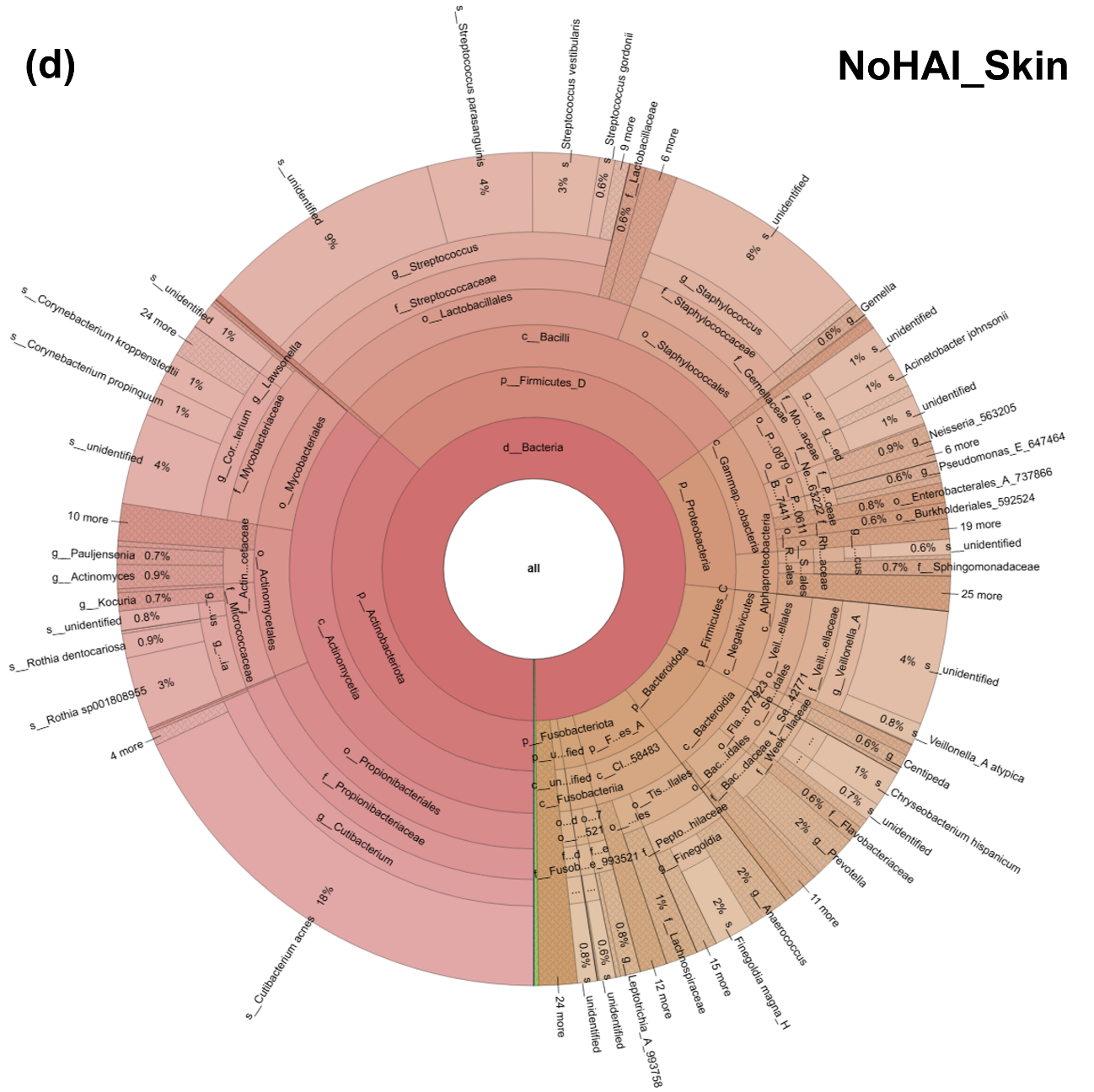


***Figure 1. (d)*** Krona chart representing the average relative bacterial composition in skin samples from the patients who did not develop an HAI after VEGs implantation (NoHAI_Skin group). HAI**,** healthcare-associated infection; VEGs**,** vascular endografts**.**
